# First Report of Crown Gall of Kiwifruit (*Actinidia deliciosa*) Caused by *Agrobacterium fabacearum* in China and the Establishment of Loop-Mediated Isothermal Amplification Technique

**DOI:** 10.3390/ijms23010207

**Published:** 2021-12-24

**Authors:** Linan He, Jinqiao Shi, Zhibo Zhao, Fei Ran, Feixu Mo, Youhua Long, Xianhui Yin, Wenzhi Li, Tingting Chen, Jia Chen

**Affiliations:** 1 Research Center for Engineering Technology of Kiwifruit, Institute of Crop Protection, College of Agriculture, Guizhou University, Guiyang 550025, China; gzhln9618@126.com (L.H.); shijq163@163.com (J.S.); zhaozhibozhi@hotmail.com (Z.Z.); RF18786614232@126.com (F.R.); gzmfx@sina.com (F.M.); xhyin@gzu.edu.cn (X.Y.); lwz9512@126.com (W.L.); gzctt126@126.com (T.C.); c18184436145@126.com (J.C.); 2 Teaching Experimental Field of Guizhou University, Guizhou University, Guiyang 550025, China

**Keywords:** crown gall, kiwifruit, *A*. *fabacearum*, LAMP

## Abstract

Kiwifruit is moderately sweet and sour and quite popular among consumers; it has been widely planted in some areas of the world. In 2019, the crown gall disease of kiwifruit was discovered in the main kiwifruit-producing area of Guizhou Province, China. This disease can weaken and eventually cause the death of the tree. The phylogeny, morphological and biological characteristics of the bacteria were described, and were related to diseases. The pathogenicity of this species follows the Koch hypothesis, confirming that *A. fabacearum* is the pathogen of crown gall disease of kiwifruit in China. In this study, Loop-mediated isothermal amplification (LAMP) analysis for genome-specific gene sequences was developed for the specific detection of *A. fabacearum*. The detection limit of the LAMP method is 5 × 10^−7^ ng/μL, which has high sensitivity. At the same time, the amplified product is stained with SYBR Green I after the reaction is completed, so that the amplification can be detected with the naked eye. LAMP analysis detected the presence of *A. fabacearum* in the roots and soil samples of the infected kiwifruit plant. The proposed LAMP detection technology in this study offers the advantages of ease of operation, visibility of results, rapidity, accuracy and high sensitivity, making it suitable for the early diagnosis of crown gall disease of kiwifruit.

## 1. Introduction

Kiwifruit (*Actinidia deliciosa*) is a berry fruit belonging to the Actinidiaceae family and it is rich in vitamin C and a variety of mineral elements [1,2]. Kiwifruit is often infected by plant pathogens, causing its food value and economic value to be seriously damaged by diseases, including soft rot, bacterial canker, and crown gall disease [3,4,5,6]. The pathogenic bacteria in *Agrobacterium* spp. can cause diseases in a wide range of hosts, among which fruit trees such as stone fruits, berries, pome fruits and nuts are more serious [7]. Once plant crown gall disease occurs, there are no effective control measures. At present, the prevention and treatment of plant crown gall disease mainly focuses on the research of physical control, pharmaceutical control [8,9], biological control [10,11,12,13,14,15,16] and genetic engineering [17,18,19,20]. Crown gall disease was recently observed in kiwifruit plants in Guiyang (Guizhou Province, China). This disease affects the absorption and transportation of water and mineral nutrients in kiwifruit, causing serious damage to plant growth which weakens the tree, ultimately resulting in the death of the tree [21]. Presently, there are few reports of kiwifruit crown gall, and the classification of these disease bacteria in China remains ambiguous. Moreover, the lack of rapid detection and the technology for early diagnosis for this disease hinders the research on its prevention and control technologies. Apart from the morphology, sequencing specific DNA regions have become a basic requirement for the accurate identification of disease pathogens. For *Agrobacterium* spp., DNA-based recognition usually relies on multi-site sequencing of 16S rDNA and the four housekeeping genes *gyrB*, *atpD*, *recA* and *rplB* [22,23,24,25]. Therefore, it is necessary to clarify the pathogen types of kiwifruit crown gall and develop an early, simple and rapid diagnostic method to detect the occurrence of kiwifruit crown gall in a timely manner so as to determine the classification status of the pathogen and prevent the occurrence and spread of this disease.

In recent years, people have developed a variety of molecular technologies, such as polymerase chain reaction (PCR), nested PCR, random amplified polymorphic DNA (RAPD) and restriction fragment length polymorphism (RFLP) technology and recombinase polymerase amplification [26,27,28]. However, these methods present disadvantages such as complex procedures, expensive reagents, time-consuming protocol and labour-intensiveness, which make it unsuitable for rapid detection in the field [29,30]. LAMP is a nucleic acid in vitro amplification technology established by Japanese scholars, Notomi et al. [31]. This method offers strong specificity, high sensitivity, rapidity, high efficiency, and accuracy [32,33]. When compared with conventional PCR, LAMP offers obvious advantages of visualisation through turbidity or colour changes [27,34,35]. LAMP technology is currently widely used for the rapid detection of pathogens such as fungi [36,37,38,39], bacteria [40,41,42] and viruses [43,44].

In this study, the pathogenic bacteria of kiwifruit crown gall disease were identified through molecular biology, pathogenicity determination, morphological observation and biological characteristics research. At the same time, a LAMP detection method for *A. fabacearum* was established to quickly detect the root system and soil of kiwifruit plants infected by *A.* *fabacearum.* The LAMP detection method can be applied for the rapid diagnosis of kiwifruit crown gall caused by *A. fabacearum* in this field.

## 2. Results

### 2.1. Disease Occurrence

**Figure 1:**
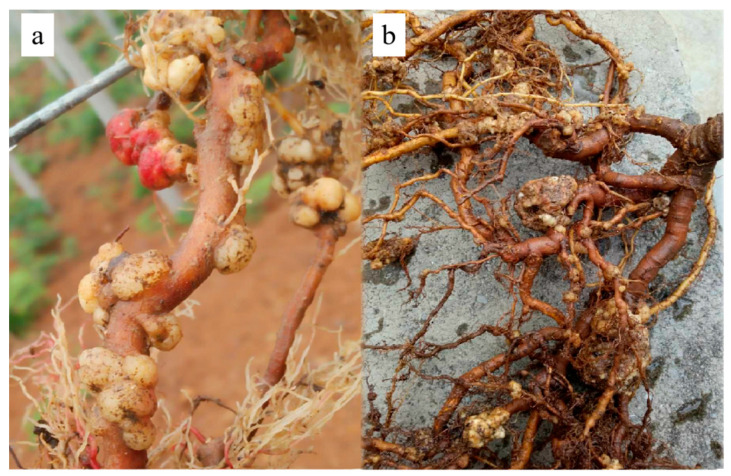
Crown gall disease in a kiwifruit plant root caused by *A. fabacearum* in the field. (**a**) Early stage of onset; (**b**) later stage of onset.

Tumours of different sizes and irregular shapes were formed mainly on the taproot system. At the beginning of this disease, spherical or nearly spherical nodules were formed on the roots, and they showed milky white, smooth surface and soft texture (Figure 1a). In the later stage, the tumour kept growing and irregular nodules were presented after the convergence of multiple tumours, with brown or dark brown colour, rough surface, hard texture and cracks developed in the middle of the tumour; the size was approximately 5~10 cm (Figure 1b). The root absorption function of severely ill plants was subsequently hindered, causing weakening of the tree vigour, reduced kiwifruit production and even plant death.

### 2.2. Pathogenicity Verification

**Figure 2:**
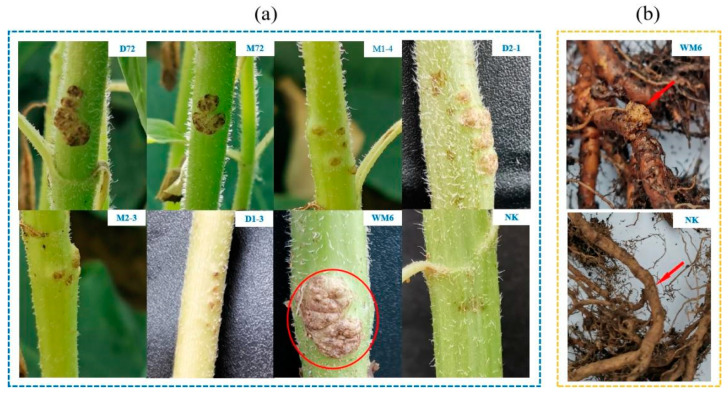
Pathogenicity test. (**a**) Symptoms of the onset of the disease one month after inoculation in sunflower plant stems; (**b**) symptoms of the onset of kiwifruit plant two months after inoculation. The strain number and control are marked.

Seven strains were isolated and used for pathogenicity verification. The pathogenicity results suggested that, after one month of inoculation, all seven strains induced tumour-like protrusions on the stem of sunflower. Among them, the strain WM6 was found to be highly pathogenic. When multiple tumours converge, they become irregular, lignified and brown (Figure 2a). Seven strains were inoculated into the roots of kiwifruit. After two months of inoculation, all seven strains induced tumour-like protrusions on the root of kiwifruit. Among them, the nodules of the WM6 strain were more obvious, and the roots of the other six strains were not obvious (Figure 2b). These symptoms, caused by artificial inoculation, were similar to those observed in actual field plantations. In the present study, sunflower and kiwifruit inoculated with sterile distilled water did not show any symptoms of crown gall. Koch’s hypothesis was met in the seven strains. Re-isolate from the inoculated plants and compare with the inoculated strains to re-isolate strains with the same colony morphology. The 16SrRNA gene sequence similarity between the re-isolated strain and the inoculated strain is 100%. The abovementioned test results conformed to Koch’s hypothesis.

### 2.3. Study on Morphological and Biological Characteristics

**Figure 3:**
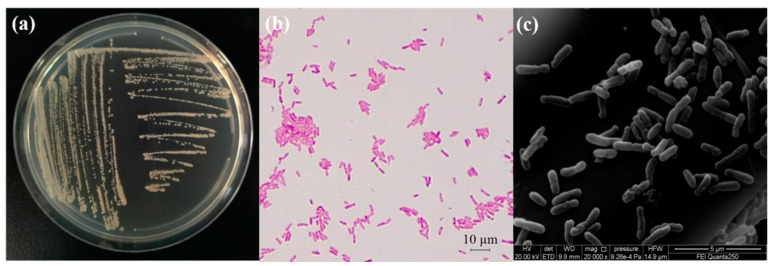
Observation of the morphological characteristics of the strain WM6; (**a**) strain colony morphology on the YEB medium; (**b**) Gram staining of the isolated strain; (**c**) scanning electron microscopic observation of the strain.

After the strain WM6 was cultured on the YEB plate for one day, the bacterial colony of the strain was observed to be round, milky white in colour, with a smooth surface, neat edges, slightly raised centre and no spores; the strain was gram-negative and rod-shaped, as observed under the scanning electron microscope. The size of the bacteria was 0.4–0.6 μm × 1.0–2.2 μm) (Figure 3).

**Figure 4:**
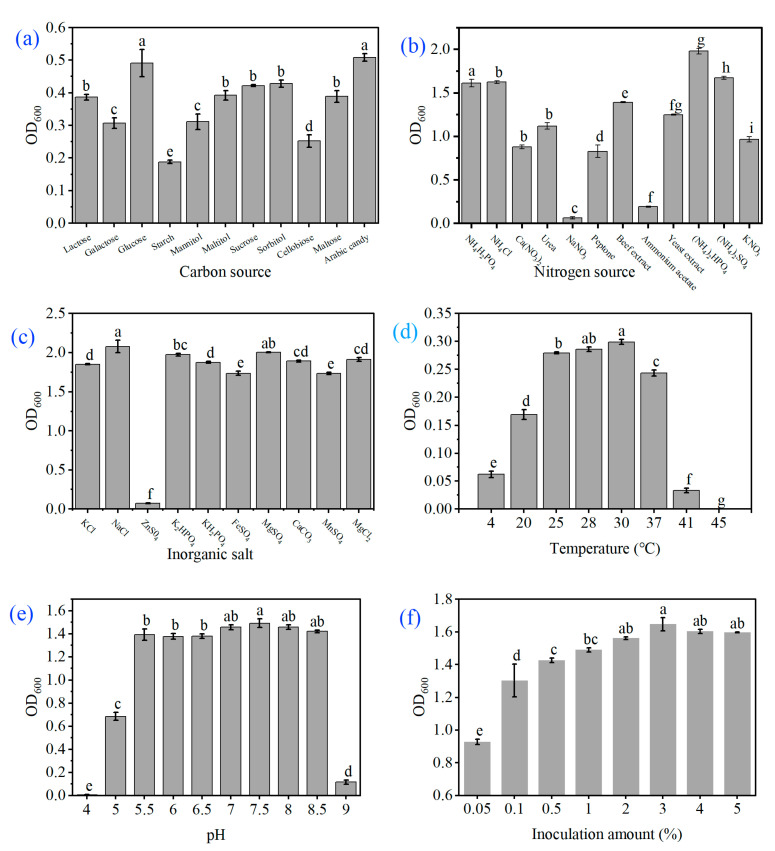
The results of the biological characteristics of the strain WM6 revealed (**a**) the effects of different carbon sources on the growth of the strain; (**b**) the effects of different nitrogen sources on the growth of the strain; (**c**) the effects of different inorganic salts on the growth of the strain; (**d**) the influence of different temperatures on the growth of the strain; (**e**) the influence of different pH on the growth of the strain; and (**f**) the influence of different inoculum on the growth of the strain.

The results of biological characterisation research revealed that the strain WM6 grew well in the fermentation media with different carbon components. We found that the strain WM6 grew faster when arabinose and glucose were used as carbon sources, which are significantly higher in concentration than other carbon sources. The OD_600_ values were 1.525 and 1.473, respectively, while the starch utilisation rate was the worst (Figure 4a); the strain showed a higher utilisation rate when the nitrogen source was diammonium phosphate. After 24 h of culturing, the OD_600_ value was 1.979, which was significantly higher than that in other treatments (Figure 4b). The utilisation effects of inorganic salts were sodium chloride > magnesium sulphate > dipotassium hydrogen phosphate > magnesium chloride > calcium carbonate > potassium dihydrogen phosphate > potassium chloride > ferrous sulphate > manganese sulphate > zinc sulphate (Figure 4c). It can grow in the range of approximately 41 °C, while its ambient growth temperature is 25–30 °C and the optimum growth temperature is 30 °C (Figure 4d); the optimum pH value of the strain is 7.5 and, at pH 4.0, the growth of the strain gets inhibited (Figure 4e). The optimal amount of inoculation was found to be 3%, followed by 4% and 5% (Figure 4f). The biological characteristics show that the most suitable carbon source is arabinose, the nitrogen source is diammonium hydrogen phosphate, the most suitable inorganic salt is sodium chloride, the optimum temperature is 30 °C, the pH is 7.5, and the inoculation amount is 3%.

### 2.4. Molecular Biology Identification and Phylogeny

**Figure 5:**
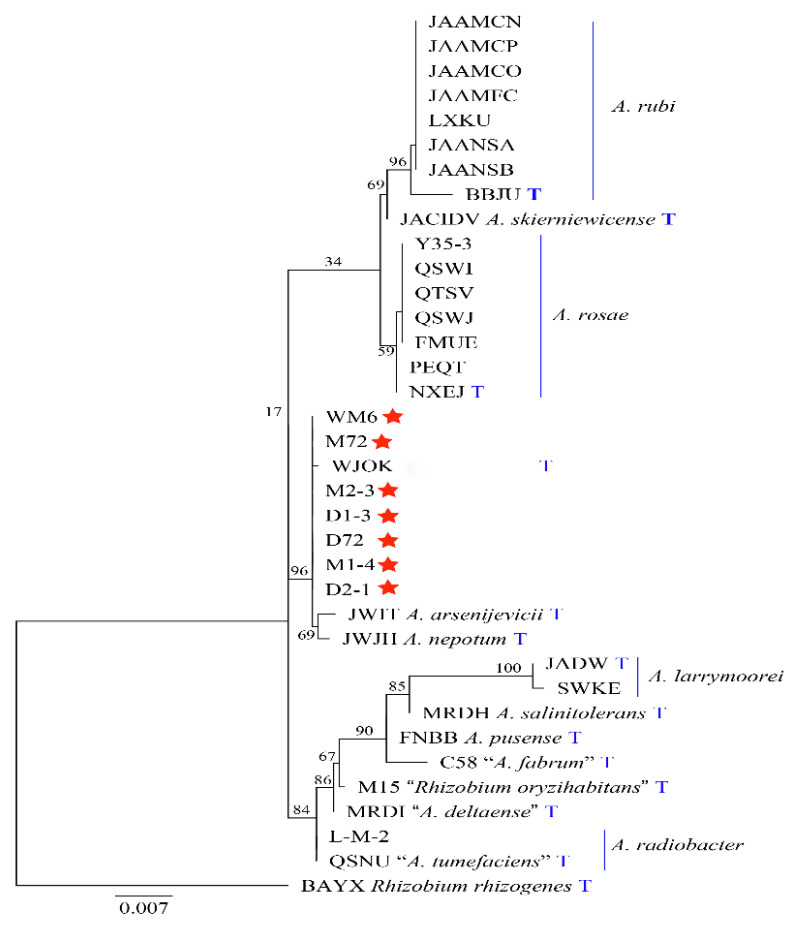
A phylogenetic tree inferred from combined analysis of 16S rDNA gene sequences for a number of species selected from *Agrobacterium* and *Rhizobium*. Bootstrap support values over 50% for maximum likelihood (ML), and the Bayesian posterior probabilities are based on 1000 bootstrap replicates. Ex-type strains are marked by an asterisk. The tree was rooted to *Rhizobium rhizogenes*. “T” stands for type strain.

**Figure 6:**
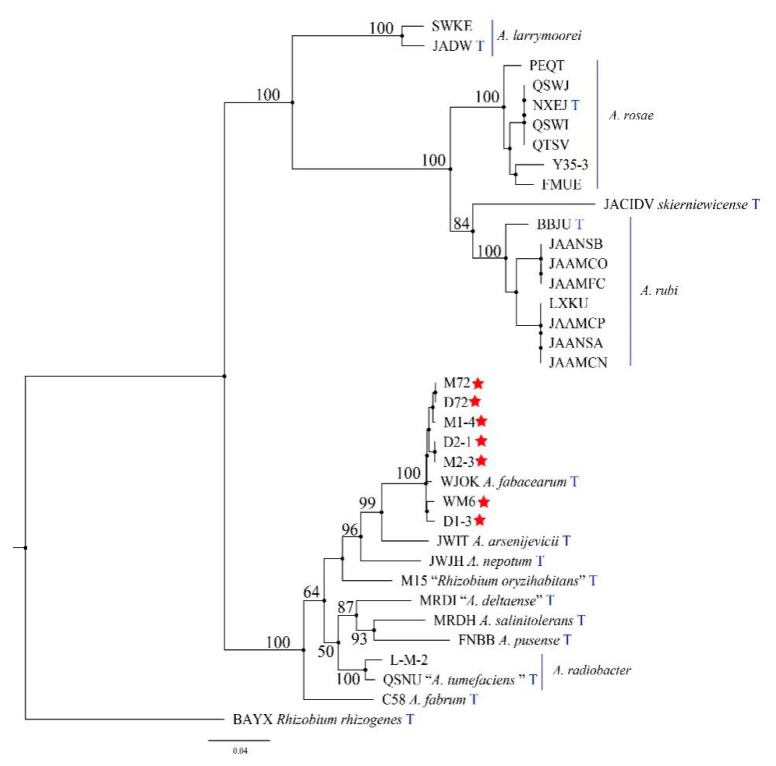
A phylogenetic tree inferred from combined analysis of *atpD-gyrB-recA-rplB* gene sequences for a number of species selected from *Agrobacterium* and *Rhizobium*. Bootstrap support values over 90% for maximum likelihood (ML), and the Bayesian posterior probabilities are based on 1000 bootstrap replicates. Ex-type strains are marked by an asterisk. The tree was rooted to *Rhizobium rhizogenes*.

**Figure 7:**
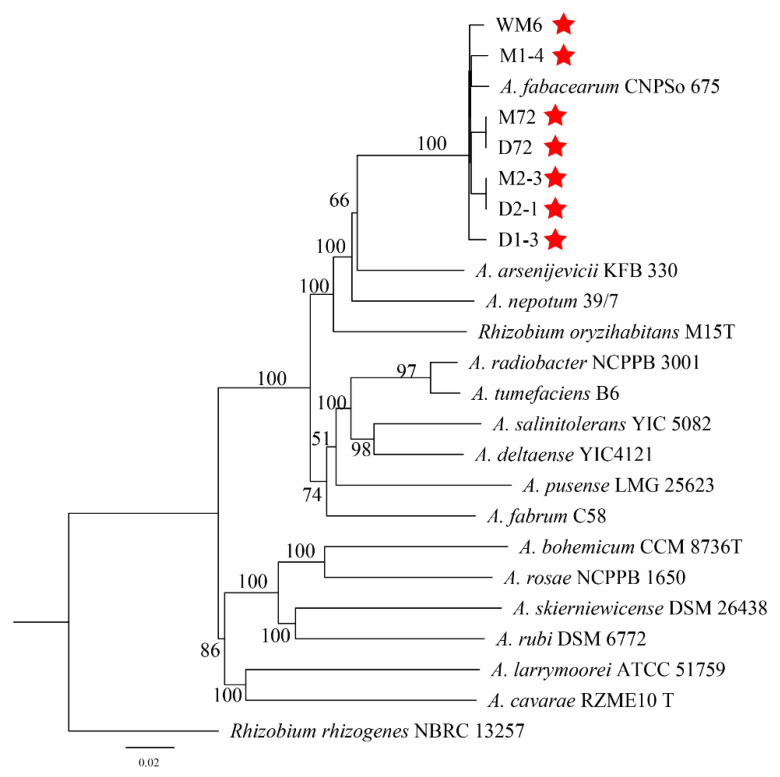
The GBDP tree (whole-genome sequence-based). Ex-type strains are marked by an asterisk.

Table 1 is used for 16S rDNA and *atpD-gyrB-recA-rplB* phylogeny analysis. Based on the phylogenetic analysis of the 16S rDNA sequence, seven strains, *A. arsenijevicii*, *A. nepotum* and *A. fabacearum* are clustered into a branch, with a support rate of 96% (Figure 5). Since the phylogeny of the 16S rDNA sequence cannot accurately distinguish the strain from the related *Agrobacterium* species, we used the MLSA of the housekeeping genes *atpD*, *gyrB*, *recA* and *rplB* to describe the target strain. The MLSA phylogenetic tree based on the tandem sequence of *atpD-gyrB-recA-rplB* revealed that, with *Rhizobium rhizogenes* as the outgroup, seven strains and *A. fabacearum* formed an independent phylogenetic lineage supported by 100% bootstrap and clustered in *Agrobacterium* spp. (Figure 6). The GBDP (genome blast distance phylogeny) tree (whole-genome sequence-based) shows that seven strains form an independent phylogenetic lineage with *A. fabacearum*, with a support rate of 100% (Figure 7).

The results of the pathogenicity verification, pathogenic bacteria 16S rDNA development tree, polygene (*atpD-gyrB-recA-rplB*) phylogenetic tree, GBDP tree (whole-genome sequence-based) and morphological characteristics research confirmed that the pathogen causing crown gall disease in kiwifruit was *A. fabacearum*.

### 2.5. LAMP-Specific Detection

**Figure 8:**
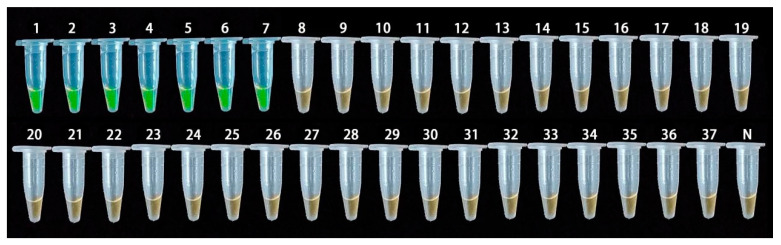
Specific detection of Loop-mediated isothermal amplification (LAMP) primer; 1–37: the PCR tube number corresponds to the DNA of the tested strain in Table 3; N: negative control.

Using the genomic DNA of the 37 test strains described in Table 2 as the detection template, the specific detection of the LAMP method was performed. The detection results are shown in Figure 8. The LAMP primer was only found to be positive for *A. fabacearum* genomic DNA, while the other control strains and negative controls were negative. The results thus confirmed that the LAMP-based *A. fabacearum* detection method proposed in this study has good specificity.

### 2.6. Sensitivity of LAMP and Conventional PCR Detection

**Figure 9:**
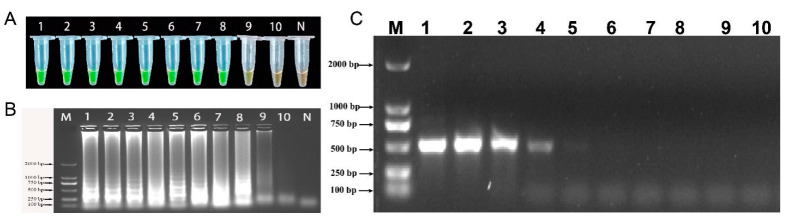
The sensitivity of LAMP and conventional PCR to detect the genomic DNA of *A. fabacearum*. (**A**) Detection by LAMP with SYBR Green I staining; (**B**) LAMP products analyzed by agarose gel electrophoresis, the LAMP product is aprox. 500 bp; (**C**): conventional PCR analyzed by agarose gel electrophoresis. 1: 50 ng/μL, 2: 5 ng/μL, 3: 5 × 10^−1^ ng/μL, 4: 5 × 10^−2^ ng/μL, 5: 5 × 10^−3^ ng/μL, 6: 5 × 10^−4^ ng/μL, 7: 5 × 10^−5^ ng/μL, 8: 5 × 10^−6^ ng/μL, 9: 5 × 10^−7^ ng/μL, 10: 5 × 10^−8^ ng/μL; M: 2000-bp DNA Marker; N: negative control.

The genomic DNA of *A. fabacearum* was serially diluted by a 10-fold gradient into 50 ng/μL, 5 ng/μL, 5 × 10^−1^ ng/μL, 5 × 10^−2^ ng/μL, 5 × 10^−3^ ng/μL, 5 × 10^−4^ ng/μL, 5 × 10^−5^ ng/μL, 5 × 10^−6^ ng/μL, 5 × 10^−7^ ng/μL and 5 × 10^−8^ ng/μL to evaluate the sensitivity of LAMP and conventional PCR. The detection limit of LAMP analysis is 5 × 10^−7^ ng/μL (Figure 9A, B), and the conventional PCR is 5×10^−^^3^ ng/μL (Figure 9C). The sensitivity of LAMP detection is 10^4^ times higher than that conventional PCR. Therefore, LAMP is more sensitive than traditional PCR.

### 2.7. Field Sample Testing

**Figure 10:**
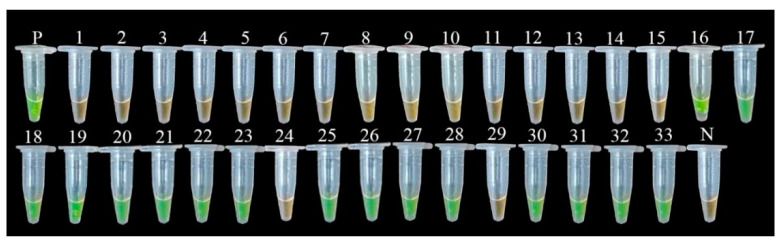
The feasibility detection of the LAMP assay. Note: 1–5: Soil DNA of healthy plant roots in the experimental plot 1; 6–11: Soil DNA of healthy plant roots in the plot 2; 12–15: Soil DNA of healthy plant roots in the plot 3; 16–17: Soil DNA of diseased plant roots in the plot 3; 18–23: Soil DNA of diseased plant roots in the plot 4; 24: control root-soil DNA; 25–28: diseased soil DNA; 29: back-linked control kiwi plant root DNA; 30–33: DNA from the root of diseased kiwifruit; P, positive control strain; N, negative control.

The established LAMP detection method was used to detect the root-soil and tissues of different plots in the main kiwifruit production area. We performed a LAMP amplification reaction on the positive control, diseased tissue, and soil genomic DNA. The reaction solution is fluorescent green (positive). A LAMP amplification reaction was performed on healthy tissues, soil genomic DNA, and negative controls. The reaction solution was orange (negative) (Figure 10). Experimental results showed that the LAMP detection method was suitable for the detection of field samples and that it can be used for the early detection of field diseases.

## 3. Discussion

Kiwifruit grows on a type of wild vine fruit tree [1]. This fruit has a high nutritional value and health care properties, which has imparted it a broad market development prospect. However, several plant diseases can significantly reduce the economic value of this plant, such as crown gall. Plant crown gall disease is a worldwide bacterial disease caused by pathogenic bacteria belonging to the genus *Agrobacterium* [45]. Morphological observation, biological characterisation and 16S rDNA phylogeny analysis were considered important for the identification of this species in the past [22]. However, owing to the variation between the isolates and the morphological overlap with other species in the *Agrobacterium* family, the morphological and biological identification of *A. fabacearum* is no longer routinely conducted. With respect to the phylogenetic markers of 16S rDNA, *recA, gyrB, atpD* and *rplB*, combined with morphological and biological characteristics, the species of *A. fabacearum* can be well identified. In the present study, the isolates from the roots of diseased kiwifruits were identified to be *A. fabacearum* based on the analysis of phylogenetic, morphological and biological characteristics, which indicated that this species has become a new threat to the survival of kiwifruit. To the best of our knowledge, this is the first report on *A. fabacearum* causing crown gall disease in kiwifruit.

Kiwifruit crown gall caused by *A. fabacearum* is a soil-borne bacterial disease. In the early stage of the disease, multiple spherical or nearly spherical nodules are formed on the lateral roots and main roots. In the later stage, the root absorption function of the diseased plants is hindered, which affects the plant growth rate and tree vigor. In severe cases, it can cause the death of the whole tree [46,47]. This event reduces the ornamental quality and commodity value of this fruit plant. In addition, the identification of pathogens based on morphology and molecular biology is time-consuming and requires high specialisation and low sensitivity, which makes it difficult to achieve rapid identification. Presently, only a few researchers have reported on kiwifruit root cancer across the world. Considering that the disease is soil-borne, early diagnosis technology is particularly important. In this study, the LAMP detection method of *A. fabacearum* was established, and the specificity and sensitivity of the detection were evaluated. The LAMP program can be used as an accurate, sensitive, and rapid alternative method for the detection of *A. fabacearum*, which can be used as a field diagnostic tool for kiwifrit crown gall disease.

The specific detection of pathogenic bacteria is of great significance for disease prevention and control [45,48,49]. In this study, 37 types of strains were used to evaluate the accuracy and sensitivity of primers. These bacteria included plant and soil pathogens, such as *A. fabacearum*, *A. rubi*, *A. radiobacter* and *A. rosae*. The designed primer set LAMP could only amplify *A. fabacearum*, indicating its good specificity. By detecting the sensitivity of LAMP, we concluded that the LAMP primer set had a high sensitivity, and the lowest detection limit reached 5 × 10^−7^ ng/μL This detection limit is lower than previously reported for the detection of potato late blight (*P. infestans*), blue mold decay (*Penicillium expansum*), maize ear and stalk rot diseases (*Fusarium temperatum*) and dried chickpea root rot (*Rhizoctonia bataticola*) [29,35,50,51]. The LAMP method established in this study is more precise and more sensitive than conventional PCR.

Past studies have shown that LAMP has been widely applied in the early detection of different pathogenic microorganisms [52,53]. In this experiment, the established LAMP technology was applied for the detection of kiwifruit root cancer samples in the field. The results showed that the genomic DNA of the diseased tissue and soil after amplification by LAMP primers was positive (fluorescent green), and all healthy samples were negative (orange). Thus, our experiment revealed that the LAMP technology can be successfully applied to the *A. fabacearum* detection of kiwi root cancer tissues and soil samples in the field. The proposed method provides strong technical support for the early diagnosis of the crown gall of kiwifruit.

## 4. Materials and Methods

### 4.1. Pathogenic Strain Isolated and Preservation

Sampling was performed from the rhizome of kiwifruit infected with crown gall in Xifeng County, Guiyang, Guizhou Province (27°2′9″ N, 106°30′38″ E). For sampling, the surface of the root was disinfected with 75% alcohol for 45 s, followed by rinsing with sterile distilled water thrice and subsequently air-drying on sterilised filter paper. The diseased tissues were cut into 2–3 pieces of dimension 2 × 2 mm and soaked in 75% alcohol for 30 s, washed thrice with sterile water, and placed in a mortar containing a small amount of sterile water for grinding until the tissues were completely ground. The inoculation ring was dipped in the grinding fluid into the D_1_M *Agrobacterium* selective medium (cellobiose 5 g, NH_4_Cl 1 g, K_2_HPO_4_ 3 g, MgSO_4_·7H_2_O 0.3 g, NaH_2_PO_4_ 1 g, malachite green 0.01 g, agar 15 g), D-1 *Agrobacterium* selective medium (mannitol 15 g, K_2_HPO_4_ 2 g, NaNO_3_ 5 g, MgSO_4_·7H_2_O 0.2 g, LiCl 6 g, bromothymol blue 0.1 g, Ca(NO_3_)_2_·4H_2_O 0.002 g, agar 15 g, pH after sterilisation was adjusted to 7.2, colour dark blue) and MW *Agrobacterium* selective medium (mannitol 10 g, NaNO_3_ 5 g, K_2_HPO_4_ 0.3 g, NaCl 2 g, MgSO_4_·7H_2_O 0.1 g, biotin 0.1 mg, 0.1% Fe-EDTA solution 2 mL, 0.1% crystal violet solution 2 mL, agar 20 g, with water added to 1 L, pH 7.0–7.2) in Petri dishes for marking and cultured in an incubator at 28 °C for 1 day. The single colony was selected and repeatedly marked on a fresh YEB medium (activated and preserved *Agrobacterium*; beef extract 5 g, yeast extract 1 g, peptone 5 g, sucrose 5 g, MgSO_4_·7H_2_O 0.5 g, agar 15 g or not, add water to 1 L, pH 7.8) plate until the colonies on the plate were completely consistent in colour, size and shape. The purified strains were selected and inoculated into a YEB liquid medium, while the bacterial liquid was prepared by shaking the culture at 28 °C at 150 rpm for 24 h. The bacterial liquid was mixed with 30% glycerol in a volume of 1:1 and stored at −80 °C until later use.

### 4.2. Pathogenicity Verification

To satisfy Koch’s hypothesis, healthy sunflower seedlings (30 days old) and 1-year-old healthy seedlings of “guichang” kiwifruit were selected for pathogenicity tests [23]. The purified strains were picked and inoculated into a YEB liquid medium and cultured on a shaker at 28 °C at 150 rpm for 24 h to prepare the corresponding bacterial suspension. A sterile needle was used to puncture the surface of the sunflower stem and the root of the kiwifruit. The punctured and injured parts were immediately inoculated in 50 μL of the bacterial suspension, while 50 μL of the sterilised distilled water was inoculated as the blank control. The experiment was repeated thrice. After treatment, the disease status of the inoculated plants was observed every alternate week, the symptoms were recorded and the photographs were taken. Koch’s hypothesis was realized by re-isolating strains inoculated on YEB medium from symptomatic plants. Morphological characteristics and 27f/149r primers were used to identify the newly isolated strains. Each experiment was repeated three times.

### 4.3. The Study of Morphological and Biological Characteristics

Morphological characteristics: The purified strains were inoculated into YEB plates by the plate streak method and the plates were cultured at 28 °C for 24 h, followed by the observation of the shape, size, colour, lustre, and transparency. According to the “Plant Disease Research Method” and “Benjamin Bacterial Identification Manual”, Gram staining was performed to observe the presence of capsules and spores, and a scanning electron microscope was used to further observe the morphological characteristics of the bacteria [54,55,56].

Biological characteristics study: the fermentation medium (composed of beef extract 3 g, peptone 10 g, NaCl 5 g and water to 1 L) was used as the basic medium to determine the carbon and nitrogen sources, contents of inorganic salts, temperature, pH and the amount of inoculum on the growth of pathogenic bacteria. Except for studying the influence of temperature on the growth of pathogenic bacteria, all other culture conditions are 28 °C dark; except for studying the influence of the inoculum amount on the growth of pathogenic bacteria, the other inoculum rates are all 1%. Each treatment was repeated thrice, and the culture was shaken at 150 rpm for 24 h, followed by the determination of the OD_600_ nm value of the fermentation broth. Carbon source: beef extract was replaced in the basic medium with glucose, maltose, starch, cellobiose, galactose, mannitol, lactose, maltitol, sucrose, sorbitol and arabinose; nitrogen source: we used NaNO_3_, ammonium acetate, peptone, Ca(N0_3_)_2_, KNO_3_, urea, yeast extract, NH_4_H_2_PO_4_, NH_4_Cl, (NH_4_)_2_SO_4_, (NH_4_)_2_HPO_4_ replaced the peptone in the basal medium; inorganic salt: ZnSO_4_, MnSO_4_, FeSO_4_, KCl, KH_2_PO_4_, CaCO_3_, MgCl_2_, K_2_HPO_4_, MgSO_4_ and NaCl replaced the inorganic salt in the basal medium; pH: we adjusted the initial pH value of the liquid fermentation broth medium to 4.0, 5.0, 5.5, 6.0, 6.5, 7.0, 7.5, 8.0, 8.5 and 9.0, respectively; temperature: 4 °C, 20 °C, 25 °C, 28 °C, 30 °C 37 °C, 41 °C and 45 °C; inoculum amount: we inoculated the seed solution 0.05, 0.1, 0.5, 1, 2, 3, 4 and 5 mL, placed at 28 °C, shaken at 150 rpm shaker for 24 h and determined the OD_600_ nm value of the fermentation broth.

### 4.4. Molecular Biology Identification and Phylogeny

Seven strains were picked to be tested on YEB medium into liquid medium respectively, followed by culturing on a shaker at 28 °C at 150 rpm for 24 h to extract the genomic DNA according to the manufacturer instruction by the Ezup Column Bacterial Genomic DNA Extraction Kit (Shenggong Bioengineering [Shanghai] Co., Ltd., Shanghai, China). The DNA products were sent to Beijing Nuohe Zhiyuan Technology Co., Ltd. (Beijing, China) for complete gene sequencing. The result of the whole gene sequence determination was submitted to BLAST in the database of the National Center for Biotechnology Information (NCBI) for homologous sequence comparison, and 16S rDNA, and *atpD*, *gyrB*, *recA*, and *rplB* sequences with similar homology species were selected for comparison. The reference nucleotide sequences of these closely related species strains were retrieved from the Gene Bank (Table 1). The website (https://mafft.cbrc.jp/alignment/server/, accessed on 17 October 2021) was used to perform single-gene sequence alignment and manually edit in BioEdit v. 7.0 as needed. BioEdit v.7.2.5 was used to combine sequence data sets. The website (http://sing.ei.uvigo.es/ALTER/, accessed on 16 November 2021) converts FASTA alignment format to PHYLIP and NEXUS format. The maximum likelihood (ML) was used to construct a phylogenetic tree. The whole gene sequence is clustered using Type (Strain) Genome Server (https://tygs.dsmz.de/, accessed on 4 December 2021).

### 4.5. Strain Source and Genomic DNA Extraction

The genomic DNA of the tested strains was extracted for specific testing. The information of the tested strains is given in Table 2. Bacterial genomic DNA extraction was performed using the Ezup column-type bacterial genomic DNA extraction kit (Sanggong Bioengineering [Shanghai] Co., Ltd., Shanghai, China) to extract the desired bacterial genomic DNA. Fungal genomic DNA extraction was performed using the Ezup column-type fungal genomic DNA extraction reagent (Shenggong Bioengineering (Shanghai) Co., Ltd., Shanghai, China) to extract the desired DNA from the strain for testing. The soil genomic DNA was extracted using the Omega Bio-tek Soil DNA Extraction Kit (Shanghai Lanbao Instrument Co., Ltd., Shanghai, China) to extract the soil genomic DNA.

### 4.6. LAMP Primer Design

**Figure 11:**
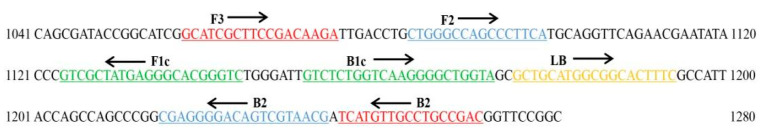
Location of LAMP-specific primers in corresponding genes.

The whole gene sequence of strain WM6 was aligned by Blast sequence, and the fragments with larger differences were detected for designing LAMP-specific primers. The sequences with different sites were saved in the txt format and the online design website PrimerExplorer V5 (http://primerexplorer.jp/e/, accessed on 27 March 2021) was used for the LAMP primer design. We obtained 5 LAMP primers, including 2 outer primers, 2 inner primers and 1 loop primer (Table 3). The position of each pair of primers and the specific sequence are shown in Figure 11. LAMP primers were synthesised by Shenggong Bioengineering (Shanghai) Co., Ltd., Shanghai, China and all primers were purified by PAGE.

### 4.7. Reaction System Establishment

The reagents were added to the 200-μL PCR tube according to the LAMP reaction system shown in Table 4. The entire reaction process was conducted on an ice bath. The PCR tube with the added reagents was placed in the L-A-320C real-time turbidimeter (Eiken Chemical Co., Ltd., Tokyo, Japan) for the reaction duration under the following reaction conditions. An L-A-320C real-time turbidity meter was used to incubate the tube at 65 °C for 60 min, followed by heat inactivation at 80 °C for 5 min. After the reaction was terminated, SYBR Green I (Shenggong Bioengineering (Shanghai) Co., Ltd., Shanghai, China) was added to the amplified product for direct fluorescent visual analysis, and the result was judged based on the colour change in the PCR tube. The fluorescent green colour of the reaction solution indicated that the sample had undergone LAMP amplification and was positive; that is, it contained the pathogenic bacteria of interest. The orange colour of the reaction solution indicated no amplification product and hence negative reaction; that is, the sample did not contain the target pathogen. Meanwhile, 5 μL of the amplified product was used for 1.2% agarose gel electrophoresis detection, and the positive amplification result showed a trapezoidal characteristic band, while the negative result showed no band amplification. Each experiment was repeated three times.

### 4.8. Specificity of the LAMP

37 bacterial strains, including strains belonging to the *Agrobacterium* genus and other non-*Agrobacterium* species, as listed in Table 2, are used for LAMP specificity. The extracted strains DNA (1 μL) were used as the template for LAMP detection. After the reaction was terminated, SYBR Green I was added to the amplified product for visual observation. The experiment was repeated three times.

### 4.9. Detection of A. fabacearum by LAMP and Conventional PCR

To evaluate the sensitivity of LAMP detection, the DNA of *A fabacearum* was extracted and used as a control for LAMP amplification for specific detection. The DNA template is diluted to a concentration of 5 ng/μL, and the DNA is diluted 10 times with ddH_2_O to make the concentrations of 50 ng/μL, 5 ng/μL, 5 × 10^−1^ ng/μL, 5 × 10^−2^ ng/μL, 5 × 10^−3^ ng/μL, 5 × 10^−4^ ng/μL, 5 × 10^−5^ ng/μL, 5 × 10^−6^ ng/μL, 5 × 10^−7^ ng/μL and 5 × 10^−8^ ng/μL, respectively, in gradient dilution DNA used as the template for LAMP detection. After the reaction was terminated, SYBR Green I was added to the amplified product for visual observation, and a 1.2% agarose gel was used for electrophoresis verification. Each experiment was repeated three times.

The conventional polymerase chain reaction (PCR) mixture contains 1μL of DNA, 1 μL of external primers (F3/B3), 10 μL of 2 × Taq PCR StarMix (Beijing Kangrun Chengye Biotechnology Co., Ltd., Beijing, China), 7 μL ddH_2_O, up to a total of 20 μL volume. The amplification program includes 35 cycles of initial denaturation at 95 °C for 2 min, 92 °C for 30 s, 56 °C for 30 s, 72 °C for 1 min, and a final 72 °C extension for 10 min. The PCR products were evaluated on 1.2% agarose gel electrophoresis in TAE buffer (1×), detected and photographed on a gel imager.

### 4.10. Test Field Samples

In order to test the effect of LAMP detection method on the detection of diseased kiwifruit roots and rhizosphere soil in the field, DNA extracted from kiwi plant roots and cultivated soils of diseased kiwifruit were collected from kiwifruit plots in the main kiwifruit producing area in Xifeng County, Guizhou Province, China (27°2′9″ N, 106°30′38″ E). Templates, and healthy roots and sterile soil as controls to determine whether LAMP analysis can detect pathogens in diseased plants. The strain WM6 of *A. fabacearum* was used as the positive control, clean water as a negative control, and SYBR Green I was used for fluorescence visual analysis of the detection results. Each experiment was repeated three times.

## 5. Conclusions

In this study, pathogenicity verification, molecular biology, morphological characteristics, and biological characteristics were used to determine the pathogen of the crown gall disease of kiwifruit to be *A. fabacearum*. This is the first report of *A. fabacearum* causing crown gall in kiwifruit. At the same time, we proposed a LAMP detection method for the rapid diagnosis of crown gall in kiwifruit caused by *A. fabacearum* in this study. We designed LAMP primers and established a LAMP reaction system to detect the specificity and sensitivity, and field samples to test and validate that the LAMP detection technology can be used as an effective tool for the early diagnosis of the crown gall of kiwifruit in the field.

## Figures and Tables

**Table 1:** Species and GenBank accession numbers of the strains in phylogeny analysis.

Species	Strain Genome Numbers
*‘A. fabrum’*	C58
*arsenijevicii*	JWIT
*‘A. deltaense’*	MRDI
*A. fabacearum*	WJOK
*A. nepotum*	JWJH
*A. larrymoorei*	JADW
*A. larrymoorei*	SWKE
*A. radiobacter*	QSNU
*A. radiobacter*	L-M-2
*A. pusense*	FNBB
*A. salinitolerans*	MRDH
*A. rosae*	NXEJ
*A. rosae*	PEQT
*A. rosae*	QSWJ
*A. rosae*	QTSV
*A. rosae*	FMUE
*A. rosae*	QSWI
*A. rosae*	Y35-3
*A. rubi*	JAAMCO
*A. rubi*	JAAMFC
*A. rubi*	JAAMCP
*A. rubi*	JAAMCN
*A. rubi*	JAANSA
*A. rubi*	JAANSB
*A. rubi*	BBJU
*A. rubi*	LXKU
*A. skierniewicense*	JACIDV
*‘Rhizobium oryzihabitans’*	M15
*Rhizobium rhizogenes*	BAYX

Note: Species with quotation marks indicate that these strains are not validly published.

**Table 2:** The species used for the LAMP specificity test.

Serial Number	Species	Sources
1–7	*A. fabacearum*	Roots of kiwifruit plant
8	*A. radiobacter*	*Amygdalus persica* L.
9	*Rhizobium rhizogenes*	*Amygdalus persica* L.
10	*A. rubi*	*Amygdalus persica* L.
11–12	*A. radiobacter*	Roots of *Prunus salicina* Lindl.
13	*A. rosae*	Roots of *Cerasus* spp.
14–15	*A. radiobacter*	Kiwifruit root system soil
16–20	*Arthrobacter* sp.	Kiwifruit root system soil
21–22	*Bacillus* sp.	Kiwifruit root system soil
23–27	*Streptomyces* sp.	Kiwifruit root system soil
28–31	*Pseudomonas* sp.	Kiwifruit root system soil
32	*Pestalotiopsis microspora*	Kiwifruit root system soil
33–34	*Phomopsis vaccinii*	Kiwifruit root system soil
35	*Trametes hirsuta*	Kiwifruit root system soil
36	*Rhodanobacter thiooxydans*	Kiwifruit root system soil
37	*Verticillium dahliae*	potato root system soil

**Table 3:** Primers used for the LAMP assays to detect *A. fabacearum.*

Primer Name	Sequence (5′~3′)
F3	GCATCGCTTCCGACAAGA
B3	GTCGGCAGGCAACATGA
FIP	GACCCGTGCCCTCATAGCGACCTGGGCCAGCCCTTCA
BIP	GTCTCTGGTCAAGGGGCTGGTACGTTACGACTGTCCCCTCG
LB	GCTGCATGGCGGCACTTTC

**Table 4:** LAMP reaction system (25 μL).

Component	Dosage	Final Concentration
10 × Isothermal Amplification Buffer	2.5 μL	1 × (Contains 2 mM MgSO_4_)
MgSO_4_	1.5 μL	6 mM (Total 8 mM)
dNTPs Mix	3.5 μL	1.4 mM each
FIP	1 μL	1.6 μM
BIP	1 μL	1.6 μM
F3	1 μL	0.2 μM
B3	1 μL	0.2 μM
LB	1 μL	0.4 μM
*Bst* 2.0 DNA Polymerase (8000 U/mL)	1 μL	320 U/mL
DNA template	1 μL	
ddH_2_O	to 25 μL	
Total reaction volume	25 μL	
Paraffin oil	20 μL	

## Data Availability

The datasets generated and/or analyzed during the study are available from the corresponding author upon reasonable request.
